# Foreword

**DOI:** 10.1007/s13280-017-0967-x

**Published:** 2017-11-24

**Authors:** Matthias Kleiner, Susanna Kaasinen

**Affiliations:** 1 Leibniz Association, Chausseestraße 111, 10115 Berlin, Germany; 2 Baltic Marine Environment Protection Commission - Helsinki Commission, Katajanokanlaituri 6 B, 00160 Helsinki, Finland

 

## This special issue and IPW from a science perspective

Phosphorus (P) is one of the most essential elements for life on earth. As fertilizer in agriculture for food and feed production and as a necessary component in industrial production, P is indispensable for human welfare. This fact is supported by the concept of the “nine planetary boundaries” (e.g., Steffen et al. 2015) which classifies the biogeochemical cycle of P—besides nitrogen flows—to the most uncertain zones with high risks.

During recent years, there has been a rising awareness of the availability and limitations of P in the Earth system as well as of its emissions and flows. Still, there are uncertainties and knowledge gaps about many aspects of the element P: its role as a major pollutant in soils and aquatic systems, P cycle processes and fluxes, management strategies for recycling, and its sufficient and efficient use.

Complex questions require interdisciplinary approaches and partnerships between science and practitioners. This was the reason, why, in 2014, the interdisciplinary Leibniz ScienceCampus Phosphorus Research Rostock (LWC Rostock) was founded as an initiative of the Leibniz Association by scientists from the Leibniz Institute for Baltic Sea Research Warnemünde (IOW), the Leibniz Institute for Catalysis (LIKAT), the Leibniz Institute for Farm Animal Biology (FBN), the Leibniz Institute for Plant Genetics and Crop Plant Research/Satellite Collections North (IPK), the Leibniz Institute for Plasma Research and Technology (INP), and the University of Rostock.

For the Leibniz Association, one of the four large non-university German research organizations, this strategic instrument is an important vehicle to promote interdisciplinary cooperation between the 91 Leibniz institutes and universities in the form of thematically focused regional partnerships highlighting relevant societal challenges.

As the president of the Leibniz Association, I congratulate the organizers of the LWC Rostock on the 8th International Phosphorus Workshops (IPW8), which took place on September 12–16, 2016 in Rostock and which can be considered as one of the most important scientific events in the field of phosphorus research in Europe.

I am very impressed about the multidisciplinarity and the high quality of the presented results of P research at this conference. This special issue will enable the free and sustainable access to information and results of the conference, and I am very sure that it will therefore contribute substantially to further enhance the P debate in the science-policy interface.

*Matthias Kleiner*

President of the Leibniz Association



## This special issue and IPW from a policy perspective

The Baltic Sea Marine Protection Commission (HELCOM) was established over four decades ago to protect the Baltic Sea environment from all sources of pollution through intergovernmental cooperation. HELCOM has ten Contracting Parties: the nine Baltic Sea coastal countries and the European Union.

Eutrophication, caused by oversupply of the nutrients phosphorus and nitrogen, is a major threat to the Baltic Sea environment. The main sources of nutrients are agriculture, waste water, and airborne emissions. The HELCOM Baltic Sea Action Plan (BSAP), adopted in 2007, aims at restoring the good ecological status of the Baltic marine environment by 2021. As reducing nutrient inputs is one of the keys to reaching this goal, the HELCOM Nutrient Reduction Scheme was established as part of the BSAP in 2007 and revised in 2013. For phosphorus, the remaining reduction target is more than 13000 tonnes of phosphorus per year to the whole Baltic Sea (Svendsen et al. 2017). The targets are allocated per country, and each sea basin has its maximum allowable input.

Many efforts to reduce nutrient inputs to the sea have been successful and good progress has been made. The input of phosphorus to the Baltic Sea has decreased by 19% when comparing the normalized average input during 2012–2014 with the average from the reference period 1997–2003. Although nutrient inputs from land have decreased considerably, the effects of these measures are generally not yet reflected in the status. Over 95% of the region was assessed as eutrophied during 2011–2015, according to the first results of the HELCOM State of the Baltic Sea report released in June 2017 (HELCOM 2017).

HELCOM relies on scientific information to tackle the complexity of the eutrophication problem. This includes, for example, assessing the eutrophication status of different sea basins and evaluating the effectiveness of measures to reduce nutrient inputs. Research is crucial for ensuring that the solution to one problem does not cause another. International scientific conferences such as the International Phosphorus Workshops (IPWs) are important for the exchange of knowledge and ideas not only among scientists but also between science and other communities. For reaching an even wider readership, it is most welcomed that the results and themes of the IPWs are shared through the conference proceedings, conference summary and conclusions, and this special issue.

Although phosphorus is often referred to as a problem in the Baltic Sea context, HELCOM recognizes its value as a vital resource and the concerns about the limited future supply of phosphorus. HELCOM Contracting Parties have agreed to enhance the recycling of phosphorus (especially in agriculture and waste water treatment) and to promote development of appropriate methodology. Furthermore, HELCOM is starting work on a regional nutrient recycling strategy. Stopping nutrients from leaking out into the sea helps solve both the problem of safeguarding nutrient supplies and the problem of eutrophication.

*Susanna Kaasinen*

HELCOM (Baltic Marine Environment Protection Commission - Helsinki Commission)
